# Liver DCE-MRI Registration in Manifold Space Based on Robust Principal Component Analysis

**DOI:** 10.1038/srep34461

**Published:** 2016-09-29

**Authors:** Qianjin Feng, Yujia Zhou, Xueli Li, Yingjie Mei, Zhentai Lu, Yu Zhang, Yanqiu Feng, Yaqin Liu, Wei Yang, Wufan Chen

**Affiliations:** 1 School of biomedical engineering, Southern Medical University, Guangzhou 510515, China

## Abstract

A technical challenge in the registration of dynamic contrast-enhanced magnetic resonance (DCE-MR) imaging in the liver is intensity variations caused by contrast agents. Such variations lead to the failure of the traditional intensity-based registration method. To address this problem, a manifold-based registration framework for liver DCE-MR time series is proposed. We assume that liver DCE-MR time series are located on a low-dimensional manifold and determine intrinsic similarities between frames. Based on the obtained manifold, the large deformation of two dissimilar images can be decomposed into a series of small deformations between adjacent images on the manifold through gradual deformation of each frame to the template image along the geodesic path. Furthermore, manifold construction is important in automating the selection of the template image, which is an approximation of the geodesic mean. Robust principal component analysis is performed to separate motion components from intensity changes induced by contrast agents; the components caused by motion are used to guide registration in eliminating the effect of contrast enhancement. Visual inspection and quantitative assessment are further performed on clinical dataset registration. Experiments show that the proposed method effectively reduces movements while preserving the topology of contrast-enhancing structures and provides improved registration performance.

Chronic liver diseases, including cirrhosis and fibrosis, are associated with high morbidity and mortality worldwide. Dynamic contrast-enhanced magnetic resonance imaging (DCE-MRI) can be used to noninvasively quantify regional and global changes in hepatic perfusion associated with liver cirrhosis and fibrosis. Changes in longitudinal intensities of the voxels in DCE-MR time-series images reflect the dynamic change in the concentration of contrast agents in tissues, which can be employed to extract quantitative parameters to quantify tumor perfusion, vascular volume, vessel permeability, and angiogenesis. A pharmacokinetic (PK) model is a widely used classical model that fits the changes in contrast agent concentration over time at each voxel^1^,^2^,^3^,^4^,^5^. However, the accuracy of PK analysis is susceptible to patient motion (e.g., inconsistent breath holding and stomach and bowel peristalsis), and such disturbance could result in voxel mis-correspondence in successive images in DCE-MRI time series. Therefore, registration of DCE-MR time-series images is a crucial preprocessing step for PK analysis and has been studied intensively in previous studies. The following factors should be considered in the registration for DCE-MR time series.

Intensity variations caused by contrast agent can corrupt the correspondence between the voxels of pre- and post-contrast images. The desired registration method should be capable of coping with intensity inconsistencies caused by contrast agent and patient motion, respectively.

As a series of images need to be aligned, the registration of DCE-MR time-series images can be considered a group-wise registration problem. New techniques of group-wise registration, such as template image selection and registration route optimization, which are ignored in most existing methods, can benefit the registration of DCE-MR time series.

Existing registration algorithms for DCE-MR time series focus on intensity variances. Because contrast-enhanced regions always expand or shrink after registration using traditional methods, several specific adjustments to the cost function or optimization method have been proposed. One example is locally rigid transformation^6^, which can penalize deviations of the Jacobian determinant of deformation^7^ to preserve the local volume. Moreover, a partial differential equation-based method^8^ using normalized gradients and a 2D/3D+time deformation model^9^ with an intensity invariance metric and constrained optimization has also been proposed. Another method has introduced a new but similar metric process to eliminate the effect of intensity inconsistencies caused by contrast agents. For instance, the Lorentzian estimator, rather than a quadratic measure, has been used to define a cost function^10^. More sophisticated similarity measurements using the PCA method^11^ or relaxation time regression error^12^ have been introduced to quantify the amount of misalignment in the time series. A third type of method integrates the PK model into the registration framework to guide the registration procedure. A previous study^13^ used the fitting error of the PK model as the similarity measure for registration. An iterative registration and segmentation method was proposed using Markov random fields (MRFs) and the PK model^14^. A similar approach^15^ used the modified Tofts model^1^,^2^,^3^ to fit enhanced frames. However, model fitting requires consideration of the characteristics and shape of each tissue and generates time intensity curves thereof, which is not practical.

Recently, several de-enhanced methods, which separate motion components from contrast enhancement by using data decomposition approaches, have been proposed and used widely. A method named progressive principal component registration (PPCR) was established^16^,^17^. In this method, images are decomposed using principal component analysis (PCA) and components with small eigenvalues are assumed to be caused by patient motion and used to iteratively guide the registration procedure. Similarly, independent component analysis (ICA) can be used to de-enhance cardiac MRI data^18^. However, the distribution of enhancement components does not satisfy the Gaussian function and its magnitude is not always small. Therefore, a more robust decomposition method is required. More recently, robust principal component analysis (RPCA)^19^ was introduced into the registration framework called Robust Data Decomposition Registration (RDDR)^20^. In this method, images were decomposed into low-rank and sparse components. The latter was assumed to be associated with contrast enhancement and was separated from the original images before registration. Only the low-rank component was used to guide the registration process. The experiment verified the effectiveness of the proposed method, which has gradually become a promising and effective technology that addresses the misalignments of DCE-MRI time-series. To date, the RDDR method^20^ has demonstrated the best performance in the liver (coronal view, breath-holds/shallow breathing) compared with the state-of-the-art methods, such as previously described PPCR^16^,^17^ and ICA^18^. However, the RDDR method is based solely on the basic group-wise registration method, which cannot ensure the rationality of deformation field. If the performance of RPCA decomposition is not suitable, large differences between frames will be generated, yielding an unrealistic deformation.

As previously mentioned, the alignment of DCE-MR time-series images should be considered a group-wise registration problem. Over the past decade, group-wise registration approaches have made substantial progress, and numerous methods have been proposed for MR brain imaging^21^,^22^,^23^,^24^. Although the core objective of group-wise registration is the construction of an unbiased atlas, which is not the key concern of DCE-MR time-series registration, many novel strategies developed for group-wise registrations, such as template image selection and registration path optimization, can be applied to address the current registration problem. For instance, a previous study^21^ assumed that all brain MR images were located on a low-dimensional manifold. Based on this assumption, the template image should be located at the center of all images with respect to the geodesic distance. Furthermore, every image should be deformed to the template image along the geodesic path. This method can effectively solve the registration problem with large anatomical variation by decomposing a large deformation between two images into a series of small deformations along the shortest path on the empirical manifold. The main advantage of this method is that unrealistic deformation can be avoided. In practice, large variations in brain MR images among different persons may result in relatively high dimensionality of the manifold and a large number of image requirements for spanning; these conditions limit the feasibility of this method. By contrast, images of the same patient are similar, except for evident intensity variations and small shape variations in liver DCE-MR time series (Fig. 1). Therefore, it is reasonable to assume that the underlying manifold has a relatively low dimensionality and the images at hand are adequate for spanning. In addition, after liver DCE-MR image assessment, we determined that image variation across time frame is somewhat smooth: that is, the variation between adjacent frames is usually small, except for certain individual frames (for example, *T*_5_ and *T*_7_. Fig. 1). Intuitively, the registration of adjacent time frame should be relatively easy. This characteristic has motivated the introduction of manifold-based group-wise registration allowing image registration to the adjacent node (adjacent time frame for DCE-MR images) and gradual deformation to the target image.

Motivated by previous works^20^,^21^, we propose a manifold-based registration framework for liver DCE-MR time series in the current study. Unlike other de-enhanced techniques for liver DCE-MRI registration, our method further applies manifold registration techniques to de-enhanced frames from RPCA decomposition. By automatic template selection on the geodesic mean and registration path optimization, the proposed method can significantly reduce deformation bias and unrealistic deformation caused by the corruption of contrast agents. The main procedure of our method is described as follows: First, liver DCE-MR time-series are assumed to be located on a low-dimensional manifold and the *k*NN graph approach is used for manifold construction. The image located at the center of the manifold based on the geodesic distance is selected as the template image. Furthermore, each image is gradually deformed to the template image along the geodesic path to decompose the larger deformation of two dissimilar images into a series of small deformations between adjacent images on the manifold. Second, RPCA is performed to separate motion components from the contrast enhancement. Only the motion component is used to guide the registration to void the effect of intensity inconsistencies caused by contrast agents. Finally, experiments in real liver datasets show that our proposed method exhibits better registration performance than state-of-the-art methods, such as RDDR^20^, particularly for the elimination of patient movements, while preserving the topology of contrast-enhancing structures.

## Method

In this section, we first introduce the RPCA method, which is used to obtain low-rank components for registration guidance. Subsequently, our registration approach is presented, which involves three steps: empirical manifold construction, template image selection, and registration path optimization. Finally, for the whole registration, we describe an iterative optimization framework, which yields a more robust registration result.

### Ethics Statements

This study was approved by the Ethics Committees of Guangdong General Hospital and Southern Medical University. All methods were carried out in accordance with the approved guidelines. Participants’ records/information was anonymous and de-identified prior to analysis. Therefore, the written informed consent of the participant was not obtained.

### Robust principal component analysis

To ensure the integrity of the proposed method, we introduce the RPCA principle and the mechanisms underlying the separation of motion components from contrast enhancement^20^. For the DCE-MR time-series 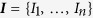, which consists of *n* time frames, a matrix *M* can be constructed to represent all dynamic frames, whose column 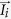 is the vectorized version of the *i*th frame 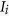. The RPCA approach aims to accurately and effectively decompose the matrix *M* with the superposition of a low-rank matrix *L* (few non-zero singular values) and a sparse matrix *S* (few non-zero entries)^19^,^25^,^26^,^27^. Such an approach is implemented by solving the following convex optimization problem:


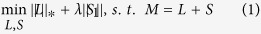


where 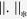 is the nuclear norm or sum of singular values, 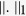 is the L1-norm or sum of absolute values, and *λ* is the trade-off parameter used to balance the contribution of the two terms. *λ* can be defined as 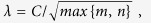 where *C* is a constant (usually equal to 1)^19^. In addition, *m* and *n* represent the rows and columns of the matrix *M*. The decomposition problem is well controlled if the low-rank component is not sparse, whereas opposite condition applies if the sparse component does not have a low-rank composition. Low-rank matrix recovery problem can be solved using several optimization algorithms, such as iterative threshold approach, accelerated proximal gradient approach, dual approach, and exact and inexact augmented Lagrange multiplier (EALM and IALM) method. In the present study, we selected IALM^28^ to solve the RPCA problem due to its accuracy, storage or memory, and complexity of computation and time conditions.

Because the variations caused by movements usually change slowly over time and affect the whole image, we can model these variations as low-rank components. By contrast, intensity variations caused by contrast enhancement typically change rapidly over time and affect only a small fraction of all pixels in an image. Therefore, we can model these variations as a sparse component, whose non-zero entries can have an arbitrarily large magnitude. In other works, by using RPCA, *M* can be decomposed as the sum of the low-rank component *L*, i.e., liver structures with movements, and the sparse component *S*, i.e., contrast changes in the time series. Only low-rank components can be used to guide the registration in reducing movements across the image series, whereas sparse components are removed to avoid the disturbance of contrast changes in the registration.

**Figure 2:**
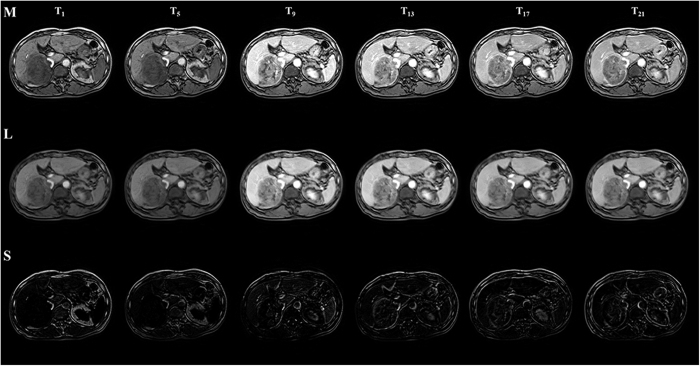
Decomposition of DCE-MR time-series images with RPCA at various time points. From top to bottom: original time series (**M**), low-rank component (**L**), and sparse component (**S**). *T*_*i*_ represents the ith time-point frame. For each data set, a total of 22 time point frames over a period of approximately 10 minutes are included, with 6 for pre-contrast and 16 for post-contrast.

Figure 2 shows the RPCA result of a liver DCE-MRI time series. It can be observed that, although the sparse component captures a part of the intensity changes caused by the contrast agent, certain residual contrast enhancement components are still observed in the low-rank component. It remains difficult to use the traditional registration method to align the low-rank component. Thus, we first propose manifold learning approaches to improve registration robustness and subsequently exploit an iterative registration framework to gradually separate intensity enhancement.

### Geodesic registration on space of empirical manifolds

Many registration methods fail because their constraints can only output a smooth deformation field but not an anatomically meaningful one. Such a problem is particularly severe for large deformation registration. To solve this problem, we define constraints on the optimization path to guarantee its optimality and rationality: (1) Anatomically meaningful intermediate nodes are learned as landmarks on the optimization path. (2) Optimization path with several intermediate nodes should be as short as possible such that it is an approximation of the optimal registration path.

Empirical manifolds, which are constructed by a graph, provide an easy way to meet these constraints. Nodes in the graph represent images, and edges correspond to the similarity between images. In this method, the manifold can represent the intrinsic similarities of original DCE-MR time series. With such manifolds and under above-mentioned constraint (1), intermediate nodes on the optimization path can be chosen from the original dataset, which can ensure anatomical meaning very well. Furthermore, considering constraint (2), nodes with high similarity will be connected to generate the minimally connected graph. Thus, the shortest connecting edge between two images can be regarded as the optimal registration path. In conclusion, empirical manifolds can help yield an anatomically meaningful and optimal path, which is defined as the shortest geodesic path.

To this end, assuming all DCE-MR time series reside on the manifold, it is relatively easier to align two nearby nodes than it is to align two distant nodes. Thus, we employ a geodesic registration method on space of empirical manifold, where each frame will only register to its nearby frame on the manifold and gradually register to the template along the geodesic path. Compared with current DCE-MRI registration method, our proposed method can yield a more robust registration, particularly for the frames far away from the template. We will introduce our registration method in the following four sections: *construction of empirical manifolds, registration path optimization, automatic template image selection and geodesic registration of DCE-MR time series.*

#### Construction of empirical manifolds

The construction of empirical manifolds assumes that all images are located on a low-dimensional manifold. In the present study, a *k*NN graph, whose nodes represent frames and edges that correspond to the similarity between nodes, is used to provide a discretized approximation of the manifolds. The *k*NN graph is constructed as follows:

1. Define the similarity between two frames. To select a metric for the construction of the *k*NN graph, two criteria should be considered. First, the measure should exhibit robustness to spatially varying intensity distortions. Second, it should be consistent with the similarity used in geodesic registration. Thus, we choose residual complexity (RC) as the metric for both *k*NN graph construction and registration, and it is used to measure the complexity of the differences in two images by using discrete cosine transformation (DCT)^29^. Compared with traditional intensity-based similarity metric systems, such as the sum of squared differences (SSD) and mutual information (MI), RC is much more robust to spatially varying intensity distortions. This is because most of traditional intensity-based similarity metric systems rely on the assumptions of independence and stability of the intensities from voxel to voxel, which are usually not satisfied in DCE-MRI due to changes in intensity in the liver and vessels^29^,^30^. To solve this problem, a previous study^29^ assumed that a spatially varying intensity field existed between corresponding images. With this assumption, the authors introduced an intensity correction field and a corresponding regularization term to adjust the cost function. To that end, the RC similarity was derived from the cost function with a DCT basis. The most significant advantage of RC is its robustness to spatially varying intensity distortions, which is also the greatest challenge in DCE-MRI registration. Furthermore, compared with local similarity measurements with high complexity, RC exhibits higher computation efficiency, which is also a significant advantage in registration. Above all, RC similarity is more suitable in both *k*NN graph construction and registration. The RC similarity 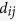 between frames *i* and *j* is defined as follows:


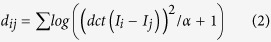


where 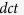
is the discrete cosine transform and *α* is the parameter used to adjust residual sparseness and is usually 0.05^29^.

2. Construct the connected *k*NN graph based on the defined similarity. The parameter *k* of *k*NN needs to be determined in practice. For a small *k* value, the graph is not connected and consists of multiple disjointed sub-graphs. For a very high *k* value, the graph becomes completely connected, and the manifold degenerates to Euclidean space. In the current study, we selected the smallest value to ensure a graph connection similar to that reported in a previous study^21^

#### Registration path optimization

Based on the constructed *k*NN graph, the geodesic distance between two nodes can be approximated using the shortest connecting paths across the graph. Concretely, the geodesic distance can be calculated by solving the following problem:


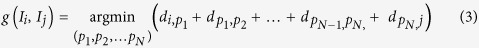


This approach identifies a series of nodes on the graph through which the node *I*_*i*_ can connect to the node *I*_*j*_ with the smallest sum of edge lengths. And *N* is the number of intermediate nodes. We call the path 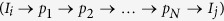 as the geodesic path between nodes *I*_*i*_ and *I*_*j*_. Eq. (3) can be optimized using Dijkstra’s^31^ algorithm or the Floyd–Warshall^32^ algorithm. In the current study, the Dijkstra’s algorithm is used for simplicity.

Frame image *I*_*i*_ can be registered to the template image *I*_*T*_ along the geodesic path, that is, *I*_*i*_ is initially transformed to its adjacent node *p*_1_ and subsequently to node *p*_2_ and so on, and finally to the template. This approach provides a method for obtaining larger deformation of two images, which can be decomposed into a series of small deformations between adjacent images on the manifold.

**Figure 3:**
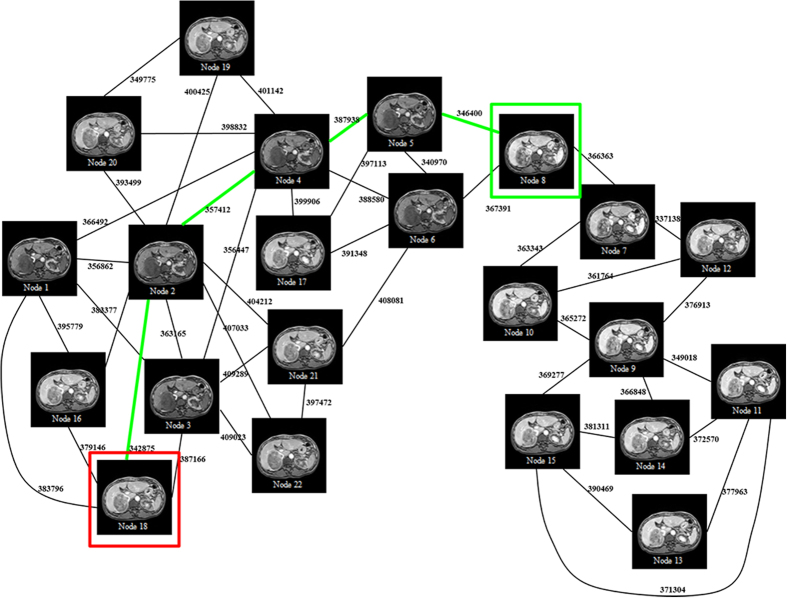
Empirical manifold of a group of DCE-MRI time frames, in which the template image is Node 8. The red box represents one frame required for registration. The green box is the template on the manifold. The green path is the geodesic path from the frame to the template.

Figure 3 is an example of the empirical manifold based on DCE-MRI time frames. The template image is Node 8, which is the frame for the eighth time point. The geodesic path of each frame to the template image can then be obtained. For example, the geodesic path from Nodes 18 to 8 (the template image) is 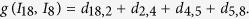


#### Automatic template image selection

For group-wise registration, computation of the template and registration route, which are least biased, is important. The original image space, namely, Euclidean space, which is the center of all training images (Euclidean mean) calculated using a linear average, is usually regarded as the template. However, the created template image is likely located far from the underlying manifold and thus lacks reasonable anatomical meaning. Herein, we use an unbiased template estimation method through geodesic averaging, rather than linear averaging. The template image *I*_*T*_ should be located on the manifold and is the center of all images according to the geodesic distance (geodesic mean):


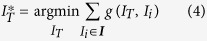


where 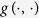 is the geodesic distance between two images on the manifold (which will be described in the next paragraph). Creating a template image directly according to Eq. (4) is not a trivial task. In practice, we select an image from ***I*** as the counterpart of 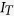 according to the following equation:


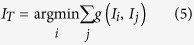


Apparently, *I*_*T*_, which is the minimum sum of the geodesic distance to all other images, is closest to the geodesic mean 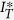.

#### Geodesic registration of DCE-MR time series

On account of contrast enhancement, the geodesic registration method cannot be directly used on original frames. Additionally, low-rank frames, in which some enhancement information has been discarded, are not sufficient for describing the intrinsic similarities in original frames. As a result, we build the empirical manifold on the original frames but compute the deformation field on low-rank frames. Deformation field will be adopted to warp original frames. Considering spatially varying intensity distortions caused by a contrast agent, we select fast free-form deformation based on b-splines^33^ with the RC similarity metric to register nearby low-rank frames on the manifold. Combining all deformation fields on the geodesic path, we can gradually warp each original frame to the template.

The procedure for clearly describing the geodesic registration of DCE-MR time series is briefly summarized follows:

1. Obtain the low-rank frames 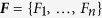 from low-rank matrix *L* (mentioned in Eq. (1)).
2. Construct the fully connected *k*NN graph from original DCE-MR time series 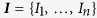.
3. Extract the geodesic path 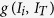 and the template *I*_*T*_ by Eqs (3) and (5).
4. Apply the results from (3) to low-rank frames, and obtain 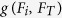 and *F*_*T*_.
5. Register each nearby low-rank frames *F*_*i*_, *F*_*i*_ on manifold by FFD+RC, and obtain the deformation field *T*_*i,j*_.
6. Combine all deformation fields on the geodesic path. As a result, deformation field from each low-rank frame *F*_*i*_ to the template *F*_*T*_ should be 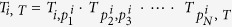, where 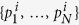 is the set of intermediate node on geodesic path 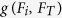.
7. Warp original frames *I*_*i*_ to the template *I*_*T*_ by the deformation field 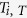.

### Iterative registration of DCE-MR time series

**Figure 4:**
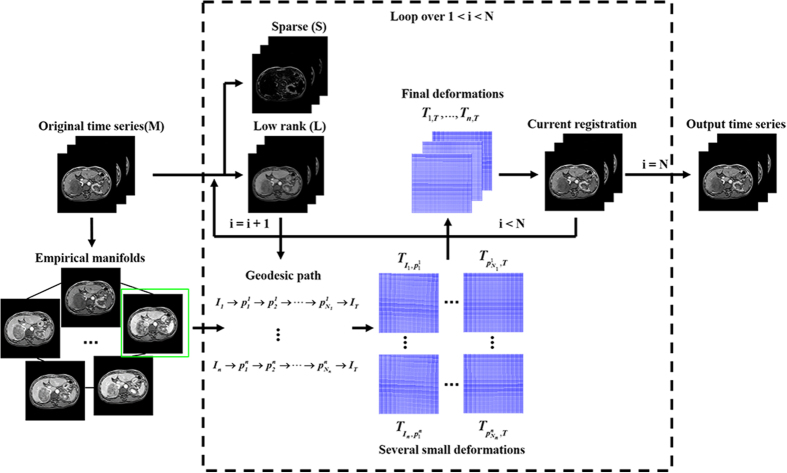
Flow chart of our proposed registration for liver DCE-MRI time-series.

Contrast enhancement cannot be completely removed using the RPCA method. Alternatively, we conduct RPCA decomposition and geodesic registration over a fixed number of iterations. In each iteration, we logarithmically increase the trade-off parameter 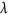, as conducted in a previously described method^20^. Our registration procedure is summarized in Algorithm 1 and Fig. 4.


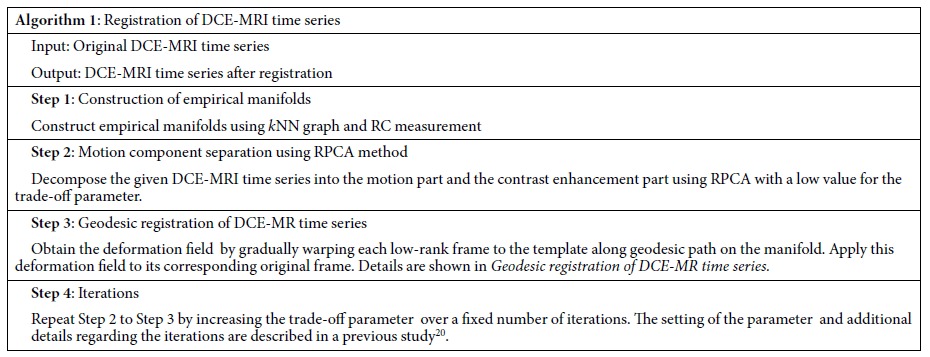


## Registration Quality Assessment

Many assessment techniques had been studied to measure the accuracy of the registration algorithm, such as the degree of smoothness of perfusion curves^34^, bi-exponential signal modeling^35^, volume changes^7^,^36^, visual inspection^6^,^10^,^16^ and time-intensity curves^16^,^17^,^20^. Here, we choose three assessment techniques: visual inspection,time-intensity curves and volume change. The computation of image similarity as a criterion is unsuitable because image similarity can be improved if contrast-enhancing structures disappear.

### Visual inspection

One criterion used to evaluate the registration algorithm is the reduction of motion artifacts when the volume loss of contrast-enhancing structures is prevented, which is crucial for the structure of liver tumors, a major concern for clinical experts. Therefore, the following visual inspection techniques are used to qualify the registration performance: (a) comparison diagram of contrast-enhancing structures in pre- and post-contrast images, (b) time-cut image for the temporal evolution of a pixel-wide line across all time series, and (c) subtraction images between pre- and post-contrast images.

### Quantitative assessment

#### Time-intensity curves

Registration accuracy can be evaluated by computing the root-mean-squared error (RMSE) between time-intensity curves (TICs) by using different registration schemes and the corresponding ground-truth (GT) TICs. A modulated sigmoid function^17^ is used to fit the signal change for visualization. Three requirements must be achieved in TICs.

Regions of interest (ROI): Clinical experts were invited to manually mark one pixel in regions that were supposed to be representative areas with contrast diffusion (e.g., tumor and tumor rims, liver rims, and vessels). Furthermore, accounting for statistical variability, nine other points around the manually marked points, which had similar contrast agent concentrations (e.g., all contrast-enhanced points or none of the contrast-enhanced points), were also automatically captured and analyzed. In summary, the region with 10 pixels was considered the ROI. Moreover, the mean ROI value was finally set as the intensity in TICs. Such an ROI can conveniently demonstrate the effect of movements on TICs.

Pseudo ground truth (GT): A pseudo ground truth was obtained by manually adjusting the position of the ROIs in every time frame on the unregistered data to best follow the feature of interest.

Vertical axis (intensity) in TICs: All intensities were normalized by dividing the baseline intensity defined in the following equation:


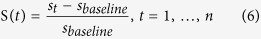


where *s*_*t*_ is the intensity of the current time point for one pixel and 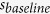 is the intensity corresponding to the pre-contrast image. *S*(*t*) represents the normalized intensity.

### Volume change evaluation

Volume changes induced by different registration methods are evaluated using the following equation:


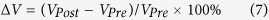


where *V*_*Pre*_ is the volume in pre-contrast frames and *V*_*Post*_ is the volume in post-contrast frames. Both volumes are measured through a manual outline of the tissue rims.

## Experiment Results

In this section, we present the registration performance for different methods through visual inspection and quantitative evaluation. For comparison, we selected the RDDR method^20^. The parameters used in RPCA and iteration process are set to be the same as those previously described^20^ for fair comparison.

### Data acquisition and computation time

Liver datasets from 11 patients were acquired (transverse view) at 1.5T using breath-hold 3D T1-weighted gradient echo pulse sequences with TR = 11 ms, TE = 3 ms, and flip angle = 15°. A total of 22 to 26 slices per patient were acquired depending on tumor size. For each slice, the pixel spacing was 1.0938 mm × 1.0938 mm and the matrix was 320 × 320. The slice thickness was 8 mm, and the slice gap was 4 mm. Patients were instructed to hold their breath at the end of expiration. A total of 22 time points were acquired, with 6 time points acquired pre-contrast and 16 time points acquired after the administration of contrast agent. The scan duration of each volume was 6–8 seconds. Therefore, over the first 14 time points, every two volumes were acquired during a breath hold. Moreover, the time interval between two breath holds was approximately 10 s. Over the last 8 volumes, only one volume was acquired in each breath hold with a 20 s break because the wash-out process is relatively slow. The whole acquisition period lasted approximately 500 s. In each dataset, five to seven interesting regions were chosen by clinical experts for the inspection of the differences in the registration scheme performance. The experiments were conducted using Matlab 2012a on an i3-3240 CPU operating at 3.4 GHz with 4 GB RAM. The entire procedure for one dataset occurred over an average of 11 h.

### Visual inspection

**Figure 5:**
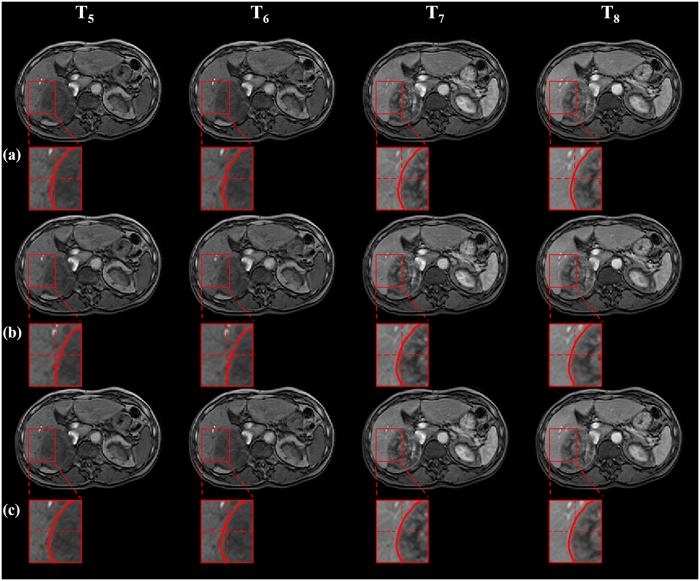
Pre-contrast and post-contrast images in a liver DCE-MR time-series of Patient 2. (**a**) before registration, (**b**) RDDR, and (**c**) our proposed method. The red box is the ROI, which represents the liver tumor. Red curve presents the tumor shape. Red cross dashed lines indicate reference lines, whose position was steady.

**Figure 6:**
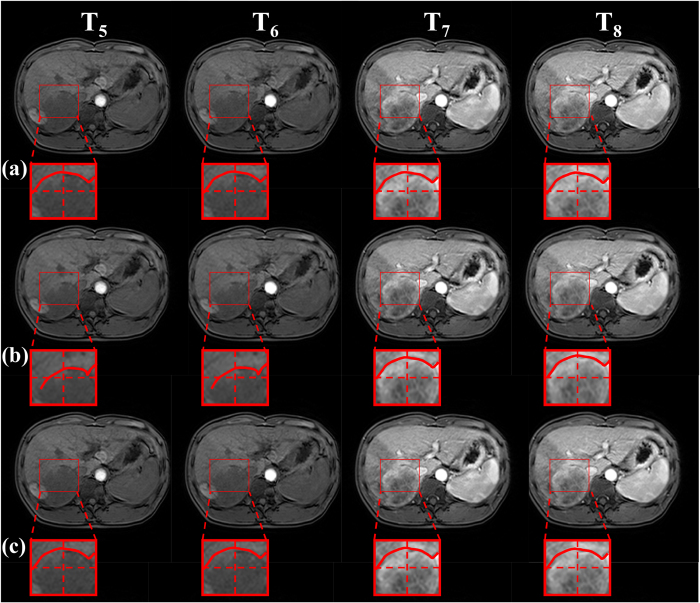
Pre-contrast and post-contrast images in a liver DCE-MR time-series of Patient 6. (**a**) before registration, (**b**) RDDR, and (**c**) our proposed method. The red box is the ROI, which represents the liver tumor. Red curve presents the tumor shape. Red cross dashed lines indicate reference lines, whose position was steady.

**Figure 7:**
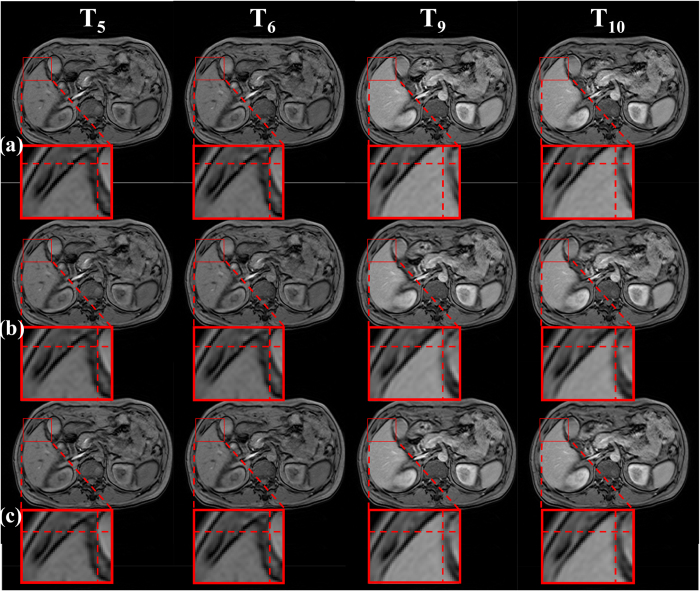
Pre-contrast and post-contrast images in a liver DCE-MR time-series of Patient 3. (**a**) before registration, (**b**) RDDR, and (**c**) our proposed method. The red box is the ROI, which represents the rim of the whole liver. Red curve presents the tumor shape. Red cross dashed lines indicate reference lines, whose position was steady.

Figures 5, 6 and 7 show the dynamic change in the contrast-enhancing structures, such as tumor and liver rims, using different registration methods. In Figs 5, 6 and 7, the first two images in each row represent the pre-contrast frames, whereas the last two images are the post-contrast frames. The red curves are the contours of the contrast-enhancing structures, on which we focused. The red crosses represent reference objects, whose position remained steady with respect to the image to observe movements or shrinkage of the contrast-enhancing structures in DCE-MR time series. Figures 5 and 6 show that the distances between the red curves and red crosses in pre- and post-contrast frames were nearly equal in the unregistered data and our proposed method (the frames in the first and third rows), whereas they changed in the RDDR method (the frames in the second row). These results demonstrated that apparent shrinks appeared in the tumor in the pre-contrast images using the RDDR method. However, tumor shape could be well preserved using our proposed method. Although the tumor volume changed in the RDDR method, the method worked well for other structures. For example, in Fig. 7, in the post-contrast frames of unregistered data, when we focused on the upper-right corner of the red box, the position of the half ellipse changed, whereas it could be corrected in both registration methods.

**Figure 8:**
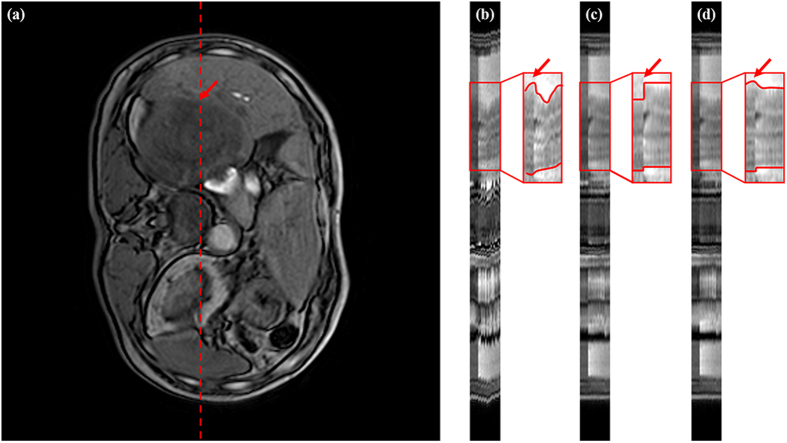
Time-cut images in a liver DCE time-series of Patient 2. (**a**) transverse plane with the tumor pointed by the red arrow. Red dashed line indicates time-cut location. Time-cut images (**b**) before registration, (**c**) RDDR, and (**d**) our proposed method.

**Figure 9:**
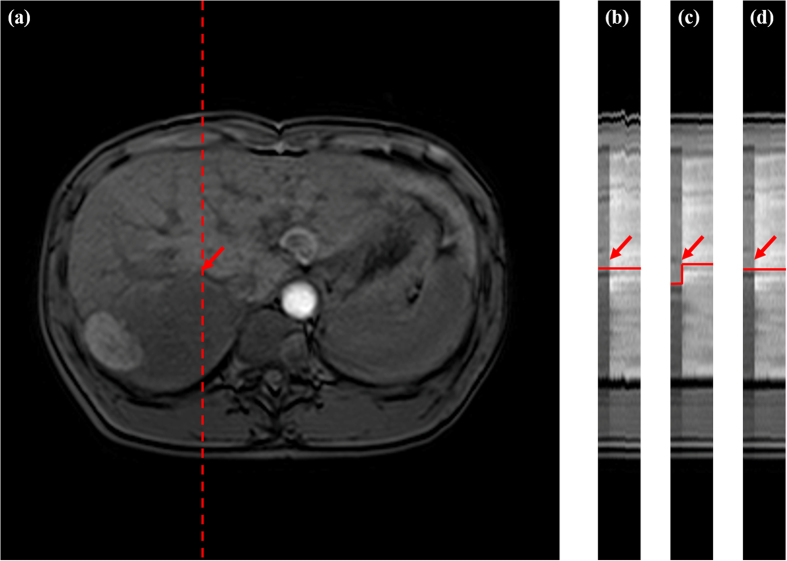
Time-cut images in a liver DCE time-series of Patient 6. (**a**) transverse plane with the tumor pointed by the red arrow. Red dashed line indicates time-cut location. Time-cut images (**b**) before registration, (**c**) RDDR, and (**d**) our proposed method.

Figures 8 and 9 show time-cut images before registration, RDDR, and our proposed method. The images were obtained from the same patients represented in Figs 5 and 6. The red dashed line in Figs 8 and 9(a) indicates the position of a pixel-wide line. The evolution of this line across all time-series using different methods can be shown in Figs 8 and 9(b–d). The red box is the liver tumor, in which the red curve describes the trend of the variations along the time line. The RDDR method could reduce most misalignments, except for those associated with the tumor. The RDDR method showed an apparent shrinkage in tumor volume in the pre-contrast images. However, our proposed method could reduce misalignments and preserve tumor volume and shape.

**Figure 10:**
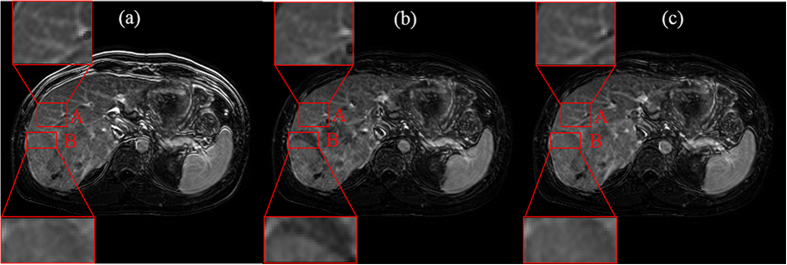
Subtraction images of Patient 8. (**a**) before registration, (**b**) RDDR, and (**c**) our proposed method. The red regions A and B are ROIs, which can show the differences among the three methods.

Figure 10 presents an example of the subtraction image. If the time series were aligned, the signal intensity of the contrast-enhancing structures, such as liver, vessels, and spleen, would also be high in the subtraction image. By contrast, the signal intensity in other non-contrast structures should be lower, for example, the signal intensity in abdominal contours. In addition, the anatomic details should be effectively visualized after registration. Figure 10 shows that both registration schemes could reduce the misalignments of the external contours. However, the vessels in region A were blurred when the RDDR method was used. The intensity of region B in Fig. 10(b) was low because of misalignments. Therefore, residual displacements appeared in these regions after registration with RDDR. Compared with the RDDR method, our proposed method demonstrated better registration performance in contrast-enhancing structures.

Notably, in this study, we focused on the registration performance with respect to tumors. However, for most of the other structures, the RDDR method performed well, although it may have performed worse than the unregistered data for liver tumors. It could be observed that external contours obtained using the RDDR method were significantly reduced (Fig. 10(b)). Moreover, the location of the whole liver was corrected, as indicated by the red crosses in Fig. 7(b). In addition, as shown in Figs 8 and 9, good performance for structures other than tumors was also obtained using the RDDR method. All of the above-mentioned examples illustrate the feasibility of the RDDR method, and our method shows better performance in tumor regions under the same parameters.

### Quantitative assessments

#### Time-intensity curves

**Figure 11:**
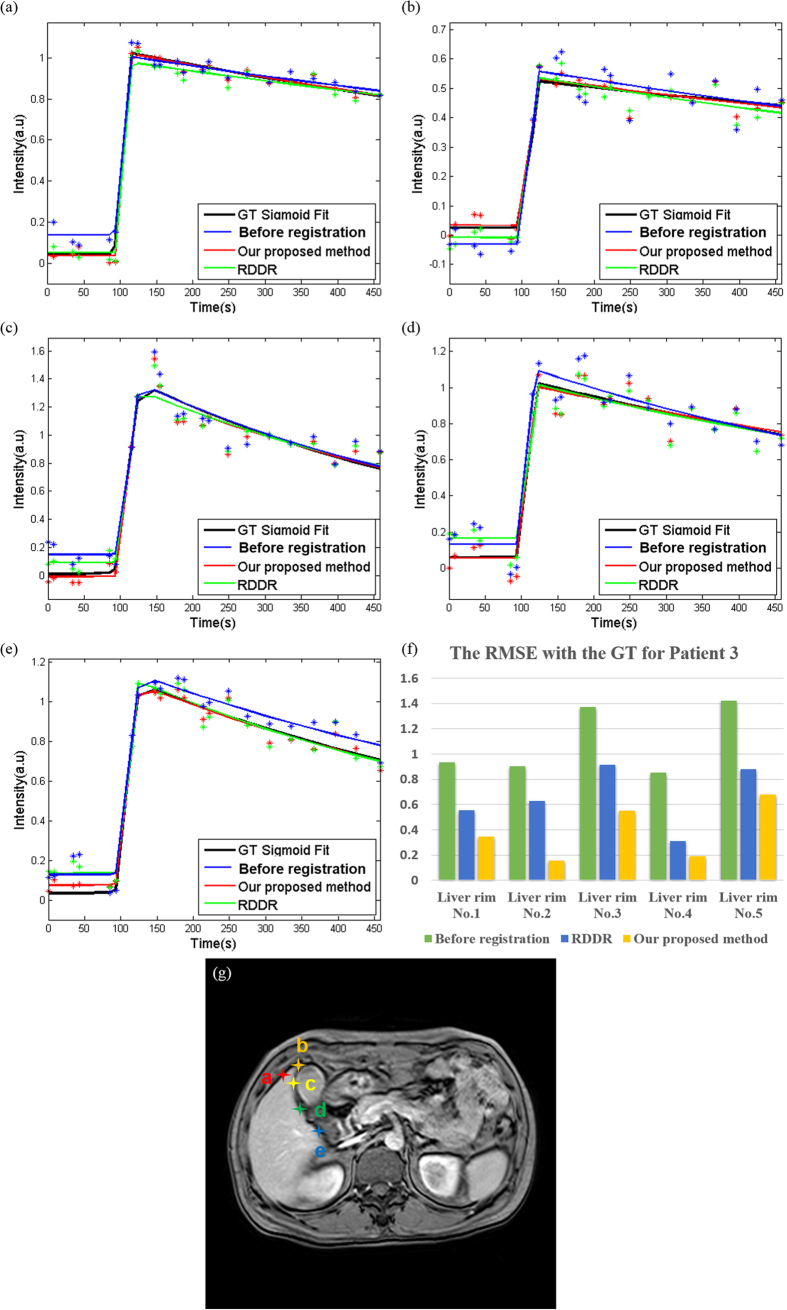
TICs for Patient 3 with different ROIs on liver rim. (**a–e**) correspond to the star-shaped marks in (**g**) and (**f**) is the RMSE with GT using different methods. The ROIs in (**a–e**) correspond to the data in (**f**) from the liver rim Nos 1–5.

**Figure 12:**
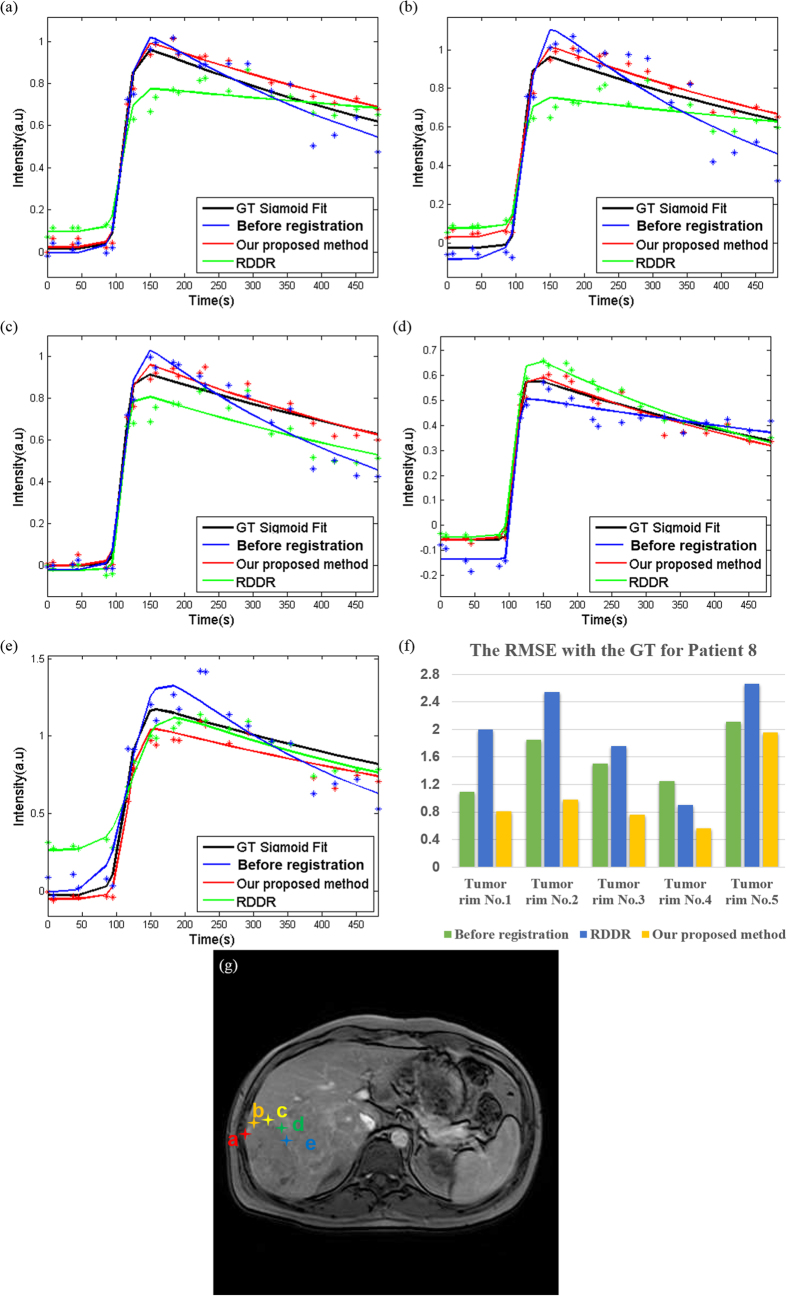
TICs for Patient 8 with different ROIs on tumor rim. (**a–e**) correspond to the star-shaped marks in (**g**) and (**f**) is the RMSE with GT using different methods. The ROIs in (**a–e**) correspond to the data in (**f**) from the liver rim Nos 1–5.

A reduction in RMSE with GT indicated an improvement in the correspondence of voxels in DCE-MR time-series. For the different ROIs in the liver rim (Fig. 11), both methods showed lower RMSE values with GT compared with the datasets before registration. In addition, the RMSE values showed better performance with respect to GT using our proposed method than that obtained using the RDDR method. However, in the case of the tumor rim (Fig. 12), RDDR showed the highest RMSE in all five ROIs, which indicated that the correspondence in the tumor rim deteriorated after registration when determined using the RDDR method. These results reflect same phenomenon, that is, the RDDR method could expand or contract the tumor volume and change the tumor shape. In addition, we also present the RMSE value with GT of all the other patients in Table 1. We observed that our proposed method presented the lowest RMSE regardless of whether the target was in the tumor rim or in the liver rim.

#### Evaluation of volume change

We suppose that the volume of liver tumors remains constant, and thus, we manually outlined the tumor volume of nine patients to evaluate changes in volume between pre- and post-contrast frames. Ideally, the volume should be similar before registration but manual measurement error may result in changes in tumor volume before registration. Thus, we also present the volume change before registration. Table 2 shows the volume changes in different methods. We observed that the RDDR method exhibited the largest volume change among all indicators. However, our method can well preserve tumor volume. In addition, the magnitude of volume change for RDDR can exclude the effects of manual outlining.

## Discussion

The main contribution of the current study is the combination of a manifold-based registration framework and an RPCA decomposition model.

For manifold-based registration, the deformation is implemented from node to node along the geodesic path, and thus, the intermediate deformed image is ensured to be located on the manifold, which is assumed as a reasonable image space. These advantages can be beneficial in preserving structural topology and can lead to a more robust registration. The proposed framework is similar to the registration for brain images reported in a previous study^21^. However, the manifold of liver DCE-MR images may have lower intrinsic dimensions because these images are acquired from the same patient. The manifold can ensure that images in one time series are sufficient for spanning, instead of requiring large numbers of samples for brain images. Therefore, the current manifold-based framework is suitable for DCE-MR time series. The experimental results verify that our approach can be effectively used for clinical liver DCE-MR data, instead of the down-sampled brain MR images reported in a previous study^21^.

As previously reported^33^,^37^,^38^, unconstrained intensity-based non-rigid registration of pre- and post-contrast images often result in a substantial volume loss of contrast-enhancing structures. To overcome these limitations, we incorporated the RDDR algorithm^20^, which utilizes RPCA decomposition to separate motion from contrast enhancement and reduces differences in contrast-enhancing structures between pre- and post-contrast images. The results presented in the current study indicate that in our liver DCE-MR time series (transverse view, breath holds), the RDDR method could actually reduce most motion and yield good performance over the entire liver, except for some contrast-enhanced structures (Figs 5, 6, 7, 8, 9, 10, 11 and 12). The volume of contrast-enhanced structures in pre- and post-contrast frames was partly altered (Tables 1 and 2). The following reasons might explain why poor registration performance was obtained in contrast-enhanced structures using the RDDR method: the data were different from the liver data previously reported^20^; and motion components in our data were relatively smaller, i.e., 5.64 mm in the tumor rim and 9.54 mm in the liver rim on average. Therefore, the variation in contrast enhancement relatively dominated the frames. Considering the principal of RPCA decomposition, the large variance in contrast enhancement would be considered a principal component regarded as a low-rank component. (In RPCA theory, the signal/low-rank component is considered to have a large variance, whereas the noise/sparse component has a smaller variance). Thus many contrast-enhancement components would be left in the low-rank component, which would not only increase differences in frames to be registered but also lead to an unrealistic deformation. This problem would be less improved by parameter adjustment and iteration. To overcome this shortcoming, the group-wise registration based on manifold was incorporated, which could significantly reduce the deformation bias.

Our results demonstrate that the proposed method can improve the registration performance of pre- and post-contrast images through shrinkage reduction in contrast-enhancing structures while simultaneously allowing for a substantial reduction in motion artifacts. In contrast to RDDR, our proposed method exhibits better registration performance (Figs 11 and 12, Tables 1 and 2).

In practice, TICs should be converted to time-concentration curves to fit the PK model. The PK model requires other complex information, such as the T1 value of tissues, arterial input function, and segmentation of different tissues and diseases. The present work only focuses on registration effects. Therefore, an accurate PK model estimation should be further studied.

The entire registration framework was designed for 2D. In our liver DCE-MR dataset, the slice thickness was 8 mm and slice gap was 4 mm. Thus, there would be less interaction between slices. Moreover, the patients were instructed to hold their breath at the end of expiration to reduce respiratory motion in the orthogonal direction as much as possible. The misalignment in the orthogonal direction of our data caused by breathing was minimized. As a result, in our data, 2D results could be an approximation of 3D results. Although this approximation was not ideal, it might be necessary to keep the computational time practical. To further accelerate the registration, we could use fewer iterations in registering each pair of nodes on the geodesic path, because the low-rank component was relatively similar after RPCA decomposition. Furthermore we could distribute the registration tasks over multiple CPUs, because the registration of one pair was independent of that of the other pairs. A compromise might be reached by one layer with its two nearby layers. Future work is necessary to develop a more accurate and efficient solution considering information between slices. In addition, the RDDR^15^ method and PPCR^12^ are also a 2D registration methods and achieve a good performance.

## Conclusion

Compared with state-of-the-art methods, such as RDDR, the proposed manifold-based registration framework for liver DCE-MR time series was demonstrated to improve registration performance, especially for liver tumors and some other contrast-enhanced structures. Thus, there is a need to adopt the manifold to ensure the rationality of deformation field, which represents the most significant advantage of our method. With the help of RPCA decomposition, frames with less contrast enhancement will be used to guide the geodesic registration. At the same time, the construction of the manifold offers a scheme for reducing registration bias, yielding an anatomically meaningful deformation. Finally, several iterations can be implemented to gradually reduce motion components. The RDDR method, which is one of de-enhanced methods, was used in this paper to separate motion from contrast enhancement. Experiments performed using all datasets demonstrated the effectiveness and robustness of our proposed method.

## Additional Information

**How to cite this article**: Feng, Q. *et al*. Liver DCE-MRI Registration in Manifold Space Based on Robust Principal Component Analysis. *Sci. Rep.* **6**, 34461; doi: 10.1038/srep34461 (2016).

## Figures and Tables

**Figure 1:**
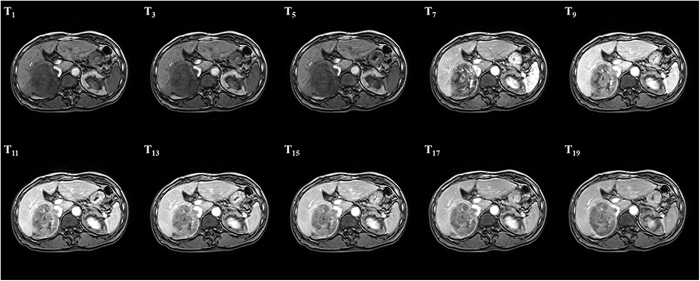
Example of liver DCE-MR time series. The number in the upper left corner represents the frame order in the time sequence.

**Table 1:** Quantitative assessment: RMSE with respect to GT for all patients.

	Before registration	RDDR	Our proposed method
Patient 1 (Tumor rim)	1.63 (1.59)	5.53 (4.28)	**1.40 (0.38)**
Patient 2 (Tumor rim)	1.50 (0.70)	2.00 (1.03)	**0.81 (0.51)**
Patient 3 (Liver rim)	0.94 (0.50)	0.63 (0.40)	**0.35 (0.40)**
Patient 4 (Tumor rim)	1.65 (0.29)	2.70 (0.63)	**1.01 (0.29)**
Patient 5 (Tumor rim)	1.68 (0.53)	1.38 (0.36)	**0.69 (0.43)**
Patient 6 (Tumor rim)	1.17 (0.68)	3.02 (2.77)	**0.73 (0.29)**
Patient 7 (Vessel rim)	1.27 (2.39)	1.97 (1.72)	**0.87 (0.70)**
Patient 8 (Tumor rim)	0.42 (0.15)	1.77 (1.23)	**0.32 (0.10)**
Patient 9 (Tumor rim)	1.25 (0.42)	1.80 (1.61)	**0.37 (0.22)**
Patient 10 (Tumor rim)	0.84 (0.56)	3.07 (3.95)	**0.43 (0.35)**
Patient 11 (Tumor rim)	0.92 (0.47)	1.72 (2.19)	**0.31 (0.20)**

Results are presented as median value (interquartile range). The optimal value is written in bold characters.

For Patient 7, five ROIs are presented, in which three are portal vein rims and the other two are hepatic veins.

**Table 2:** Evaluation of volume change between pre- and post-contrast frames (in %) for nine patients.

	Min	Max	Median	Upper quarter	Lower quarter
Before registration^b^	−3.20	1.47	−0.33	0.70	−0.83
Our proposed method	−4.59	4.26	−0.23	1.73	−1.62
RDDR	−12.50	50.21	15.78	19.84	7.88

Results are presented as min, max, median, upper, and lower quarter.

^a^Volume change before registration is also illustrated because of manual measurement error.

^b^Two patients showed no apparent tumor.
